# Synaptic self-organization of spatio-temporal pattern selectivity

**DOI:** 10.1371/journal.pcbi.1010876

**Published:** 2023-02-13

**Authors:** Mohammad Dehghani-Habibabadi, Klaus Pawelzik

**Affiliations:** Institute for Theoretical Physics, University of Bremen, Bremen, Germany; Research Center Jülich, GERMANY

## Abstract

Spiking model neurons can be set up to respond selectively to specific spatio-temporal spike patterns by optimization of their input weights. It is unknown, however, if existing synaptic plasticity mechanisms can achieve this temporal mode of neuronal coding and computation. Here it is shown that changes of synaptic efficacies which tend to balance excitatory and inhibitory synaptic inputs can make neurons sensitive to particular input spike patterns. Simulations demonstrate that a combination of Hebbian mechanisms, hetero-synaptic plasticity and synaptic scaling is sufficient for self-organizing sensitivity for spatio-temporal spike patterns that repeat in the input. In networks inclusion of hetero-synaptic plasticity that depends on the pre-synaptic neurons leads to specialization and faithful representation of pattern sequences by a group of target neurons. Pattern detection is robust against a range of distortions and noise. The proposed combination of Hebbian mechanisms, hetero-synaptic plasticity and synaptic scaling is found to protect the memories for specific patterns from being overwritten by ongoing learning during extended periods when the patterns are not present. This suggests a novel explanation for the long term robustness of memory traces despite ongoing activity with substantial synaptic plasticity. Taken together, our results promote the plausibility of precise temporal coding in the brain.

## Author summary

Neurons communicate using action potentials, that are pulses localized in time. There is evidence that the exact timing of these so called spikes carries information. The hypothesis, however, that computations in brains are indeed based on precise patterns of spikes is debated, particularly because this would require the existence of suitable detectors. While theoretically, individual neurons can perform spike pattern detection when their input synapses are carefully adjusted, it is not known if existing synaptic plasticity mechanisms indeed support this coding principle. Here, a combination of basic but realistic mechanisms is demonstrated to self-organize the synaptic input efficacies such that individual neurons become detectors of patterns repeating in the input. The proposed combination of learning mechanisms yields a balance of excitation and inhibition similar to observations in cortex, robustness of detection against perturbations and noise, and persistence of memory against plasticity during ongoing activity without the learned patterns. The proposed learning mechanism enables groups of neurons to incrementally acquire sets of patterns thereby faithfully representing their ‘which’ and ‘when’ in sequences. These results suggest that computations based on spatio-temporal spike patterns might emerge without any supervision from the synaptic plasticity mechanisms present in the brain.

## Introduction

Despite decades of research, it is still debated which coding schemes are used in central nervous systems. While in early sensory areas of cortex, stimuli appear to be represented mostly by spike rates, it cannot be disputed that temporal information is faithfully processed. In fact, experimental studies on visual, auditory, olfactory, and somato-sensory cortex indicate that neurons can respond rather deterministically to inputs, underlining the possibility of precise spike codes. [1–8]

While this can in principle be achieved by modulated spike rates in large populations of neurons it is tempting to hypothesize that at least in higher areas, as, i.e., frontal cortex, temporally precise responses of individual neurons play an important role.

A range of theoretical studies attempted to elucidate mechanisms that could support precise coding of spatio-temporal patterns [9, 10]. It was found that with suitable synaptic weights, even simple integrate-and-fire neurons are sensitive to specific spatio-temporal input spike patterns. For instance, the Tempotron [9] was introduced as an extension of the Perceptron [11] to perform classification and detection of spatio-temporal patterns with a spike response to patterns only from a given set with a supervised algorithm for potentiating and depressing a neuron’s afferents. The number of patterns that a neuron can learn to classify depends on their length, the time constants of the neurons and the synaptic inputs [12]. While in the Tempotron the action potential is allowed to occur anywhere during the time of the learned patterns, it was later shown that neurons can be forced to fire also at a specific time [10, 13] during a specific pattern’s presence, which can be achieved by several more or less realistic synaptic mechanisms [14, 15]. Both the Tempotron and the Chronotron employ supervised learning mechanisms based on label and time, respectively.

Supervised learning of spatio-temporal patterns seems at odds with reality, where the input is not labeled, impinges on the neuron continuously, and is subject to distortions and noise. In particular, it would need to explain how synaptic plasticity mechanisms become informed which aspect of the data should be taken into account when a label comes only long after the patterns. A recent study addressed this latter problem. It showed that neurons can recognize spatio-temporal patterns embedded in a background of noise using only weak supervision where the known number of repetitions of a pattern is used for optimizing synaptic efficacy [16]. Based on the N-methyl-D-aspartate (NMDA) receptor [17–19], a learning rule was proposed [16] that yields similar results as obtained by optimization. The biological plausibility of this correlation-based rule, however, is questionable. It does not respect Dale’s rule since synapses can change their sign, it is still supervised, and it requires a careful selection of potential weight changes such that only a given small percentage of potential changes become effective.

Therefore, it remains an open question if existing mechanisms of synaptic plasticity can be identified which enable neurons to specialize on statistically dominant patterns in the temporal stream of their inputs in an entirely unsupervised manner.

We use basic Hebbian mechanisms for the plasticity of both, excitatory and inhibitory neurons which for excitatory synapses resemble the NMDA-receptor. Dale’s law is enforced, i.e., inhibitory and excitatory neurons can not change into one another throughout the learning process.

The instability of Hebbian mechanisms for excitatory synapses is contained by a combination of three known mechanisms. First, upper bounds on synaptic efficacies are imposed. Second, we implement synaptic scaling: It has been shown that neurons do not remain silent for long periods, but scale their weights to achieve a genetically intended spike rate [20]. Third, we employ (post-synaptic) hetero-synaptic plasticity which provides negative changes of efficacy (i.e. long term depression; LTD) through by a competition for resources for weight increases that are provided by the post-synaptic neuron (e.g. receptors).

When several target neurons are present, we also include pre-synaptic hetero-synaptic plasticity, which induces competition for resources provided by the pre-synaptic neuron. This mechanism serves specialization of target neurons in subsets of all patterns [21].

The combination of these realistic mechanisms turns out to be sufficient for the self-organization of pattern detection in single neurons. At times when no pattern is present excitatory and inhibitory inputs become globally balanced. During the time when a learned pattern is present we find detailed balance, where excitatory and inhibitory inputs cancel each other in temporal detail. These results parallel findings of the global and detailed balance of excitation and inhibition in cortex [22–24].

The resulting synaptic efficacies are then shown to ensure robust pattern recognition that is resistant to temporal jitter and noise. When basing learning on jittered patterns and also on Poisson rate modulations instead of precisely repeating patterns we obtain even more robust pattern detection. These results underline that the proposed mechanisms can be based on imprecise patterns and temporally modulated rate codes.

Next, we wondered if and how learned memory traces vanish during ongoing plasticity when only random patterns are presented which contain no statistically dominant structures. We find extremely long memory persistence already for a single output neuron. This leads to the question if the proposed mechanisms might contribute to solving the stability-plasticity problem [25], such that they would support incremental learning where patterns occur rarely and are intermingled with random activity and/or different patterns. We investigated this for groups of output neurons where competition for patterns is induced by pre-synaptic hetero-synaptic plasticity [26]. Thereby the output neurons specialize on different subsets of patterns such that the group as a whole self-organizes faithful representation of the ‘which’ and ‘when’ of patterns in the input. The memory persistence in this setting is finally shown to support incremental learning of sets of patterns in neuronal populations.

## Results

In all simulations, we consider simple leaky integrate and fire neurons [27, 28] with a fixed membrane time constant of 15 ms and pre-synaptic spikes originating from 500 input neurons (80% excitatory and 20% inhibitory). They provide input currents via kernels that have the shape of alpha-functions. For each synapse, the amplitudes of the input currents depend on a single parameter, the synaptic weight. The kernels have different time constants for excitatory and inhibitory synapses. The signs of the weights are not allowed to change during learning, i.e., Dale’s law is enforced.

### Single post-synaptic neuron

Synaptic plasticity is based on correlations between the input-kernels and deflections of the membrane potentials with respect to a threshold. For changes of the inhibitory synapses, positive deflections increase the weights, and negative deflections lead to their decay. For changes of the excitatory synapses, we let only positive deflections contribute, mimicking NMDA-dependent mechanisms. Without further constraints, this Hebbian mechanism is unstable for excitatory efficacies.

Runaway instabilities are avoided by a combination of three simple but biologically highly plausible mechanisms: First, unbounded growth is made impossible by clipping the weights at upper limits for excitatory synapses. Second, positive weight changes for excitatory synapses are quenched when the long time activity exceeds a pre-determined rate, which mimics synaptic scaling [20, 29]. Third, the negative weight changes for excitatory synapses are induced by hetero-synaptic plasticity. Specifically, we include post-synaptic hetero-synaptic plasticity where the weight changes of different afferents are made dependent such that the resources needed for strong increases of the post-synaptic contributions to synaptic efficacies are taken from synapses which would otherwise increase only weakly. Thereby the latter synapses’ efficacies become reduced. Note, however, that this does not imply strict normalization of excitatory weights (see Materials and methods).

**Fig 1:**
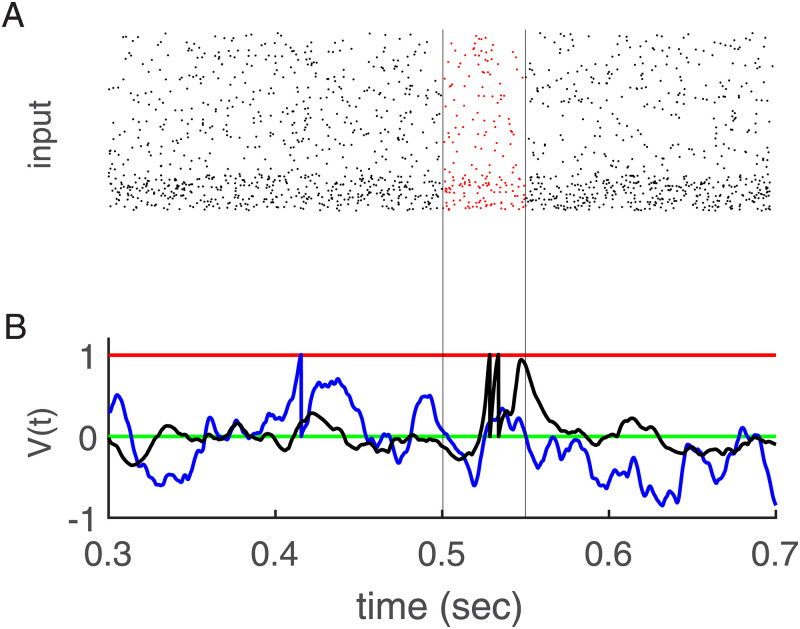
Learning of one embedded pattern. **(A)** Input activity in the raster plot. Five hundred afferents (80% excitatory and 20% inhibitory) send inputs to one post-synaptic neuron. The excitatory neurons’ rate is 5 Hz, and the inhibitory neurons’ rates are 20 Hz. There is a random embedded pattern of length 50 ms in the red area between black vertical lines. **(B)** Membrane potential versus time. The black trace shows that after learning the fluctuations outside of the embedded pattern are attenuated as compared to the case where the weights were learned from random background alone. As result two spikes occur during embedded pattern time (between the vertical black lines). The green line is for resting potential, red is for threshold, blue is membrane potential after weight initialization by learning with only random patterns, and black is for after learning.

It turns out that these ingredients are sufficient for robust self-organization of spatio-temporal spike pattern detection in single neurons. As an example, Fig 1 shows the membrane potential (MP) of a single neuron before and after learning a random pattern of length 50 ms that has the same statistics as the random background but repeats in every training epoch of length 1000 ms.

**Fig 2:**
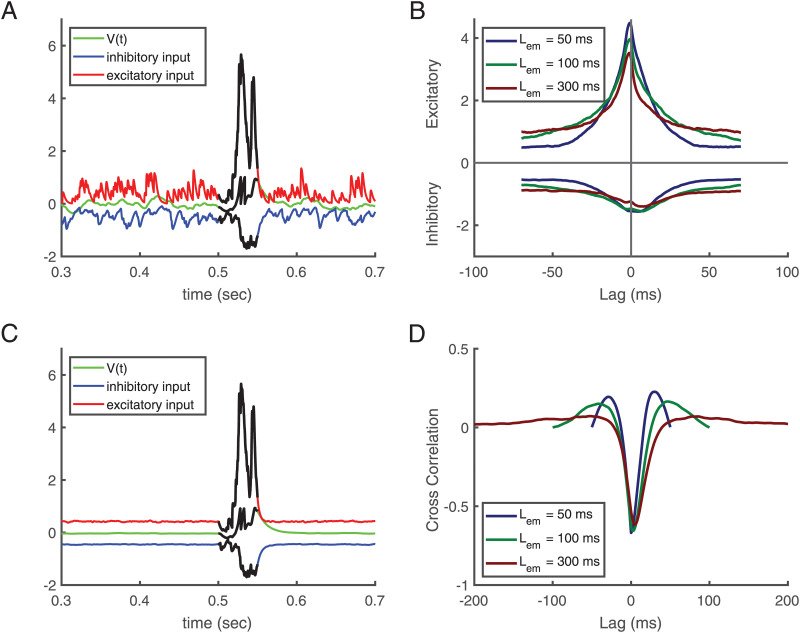
Balance of excitation and inhibition. **(A, C)** A fixed repeating pattern is embedded between 500 ms and 550 ms, i.e. duration *L*_*em*_ = 50 ms. **(B, D)** Averages over different learned patterns of lengths *L*_*em*_ = 50, 100, and 300 ms starting from 500 ms (using 500 epochs). **(A)** Excitatory and inhibitory inputs and the membrane potential after convergence of self-organization with this pattern. **(B)** Spike triggered average of respective inputs for different pattern lengths after convergence. In the more extended patterns the minimum of inhibitory inputs follows the maximum of excitatory inputs. **(C)** Average contribution of excitatory and inhibitory inputs from 500 epochs. **(D)** Cross-correlation between inhibitory and excitatory inputs for neurons that where exposed to learned patterns. In all figures, the length of the training epoch is 1000 ms, and the desired number of spikes is 2, i.e., the desired firing rate is *r*_0_ = 2 Hz.

Fig 2 captures the combined effect of the plasticity mechanisms on the inputs to a post-synaptic neuron. After convergence of the weights, we separated the excitatory and inhibitory inputs to see how their respective contributions lead to firing only during the pattern (Fig 2A). In particular, the antagonistic effect of correlations based Hebbian plasticity of excitatory and inhibitory synapses leads to global and detailed balance. Fig 2A indicates that inhibitory and excitatory inputs cancel each other outside of the embedded pattern in the mean (global balance). Averaging these inputs on epochs (Fig 2C) explicitly shows that in the mean fluctuations are removed, which corresponds to global balance. In contrast, during the time of the embedded pattern, the respective contributions to the membrane potential both increase but remain mostly balanced also across time (detailed balance). This balance is the fixed point of the weight dynamics. Fig 2B depicts the spike-triggered average of the inhibitory and excitatory inputs, respectively, underlining the detailed balance during the learned pattern. In fact, only some residual imbalance between excitatory and inhibitory afferents allow the post-synaptic neuron to fire during this period of time. The detailed balance during the embedded pattern is quantified by the anti-correlation between excitatory and inhibitory afferents which is substantial and minimal close to zero time shift (Fig 2D). Last not least, the synaptic scaling enforces the desired mean firing rate of 2Hz leading to two spikes during the pattern. Thereby, synaptic scaling limits the growth of total excitation which additionally is constrained by clipping synaptic weights at a maximal value. Note that in this model pre-synaptic hetero-synaptic plasticity is the only source of LTD for excitatory synapses.

To quantify the performance of the learning mechanism for ensembles of random patterns, we consider the average percentage *R* of spikes that correctly detect the pattern:

$$\begin{array}{c} R={\left\langle \frac{n_{s}^{p}\left(L\right)}{n_{s}+\zeta }\right\rangle }_{\mu }, \end{array}$$ (1)

where *n*_*s*_ is the total number of spikes during a testing period and $n_{s}^{p}$ the spikes’ number related to the presence of the pattern to be detected. Since patterns can induce spikes also shortly after the pattern due to the finite decay time of the excitatory synaptic kernel, the time window for testing if spikes occur inside the pattern is extended by *L* ms after the pattern has ended. Adding an arbitrary small number *ζ* to the denominator ensures a definite result (*R* = 0) when no spikes occur at all. The ratio is averaged for an ensemble of independent embedded patterns *μ*.

Occasionally, we also consider a variant of this criterion R* where the average is taken only on the epochs in which there is at least one spike occurring in post-synaptic neuron:

$$\begin{array}{c} R^{*}={\left\langle \frac{n_{s}^{p}\left(L\right)}{n_{s}}\right\rangle }_{\hat{\mu }}, \end{array}$$ (2)

where $\hat{\mu }$ refers to the ensemble of independent embedded patterns for which the post-synaptic neuron elicited at least one spike during the pattern presentation.

**Fig 3:**
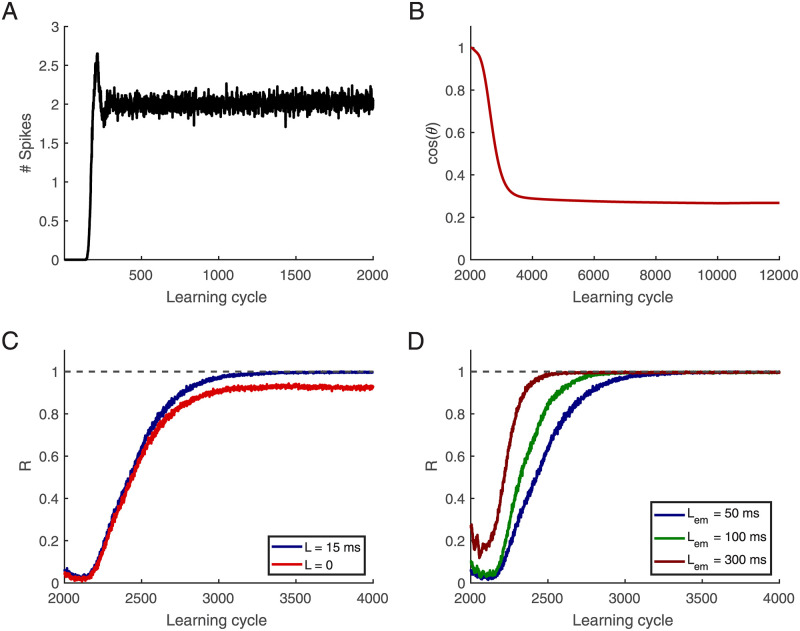
Convergence of learning. There is no embedded pattern till learning cycle 2000 in afferents, and there is a 50 ms embedded pattern in afferent from learning cycle 2001 shown in afferents beginning from 500 ms. **(A)** Number of elicited spikes versus learning cycle, on average the post-synaptic neuron fires two times in response to the noisy background. **(B)** The cosine between the current weight vector and the initial weight vector at learning cycle 2000. **(C)** Learning performance R versus learning cycle, for *L* = 0 and *L* = 15 ms. A 50 ms pattern is embedded between 500 and 550 ms. **(D)** R versus learning cycle, *L* = 15 ms. Patterns duration, *L*_*em*_, are 50, 100, and 300 ms, shown in afferents from 500 ms. R is an average of 500 simulations, in which there are 500 afferents, and the length of the training epoch is 1000 ms. The desired firing rate is *r*_0_ = 2 Hz.

Before embedded patterns are shown in this study’s simulations, only random spike patterns are shown; thus, postsynaptic neurons have already achieved *r*_0_ mainly because of synaptic-scaling. Fig 3A depicts the number of spikes in a single postsynaptic neuron at each learning cycle with only random input spike patterns. There are no output spikes in the first learning cycles since weight vectors are small in magnitude. However, on average, the neuron learns to fire at *r*_0_ Hz after a few learning cycles. Then we show an embedded pattern in each cycle after 2000 learning cycles and calculate the cosine between the weight vector at learning cycle 2000 and the new weight vector (Fig 3B). While the neuron has achieved *r*_0_ at learning cycle 2000, the weight vectors are changed when the embedded patterns start to be shown. Fig 3C shows that neurons learn to fire only in response to the embedded pattern and remain silent otherwise. Extending the testing window by *L* = 15 ms reveals that the performance becomes perfect. Fig 3D shows that using longer embedded patterns allows neurons to learn them faster: a more extended embedded pattern leaves more room for residual excitatory-inhibitory imbalances and more contribution to the weight changes; therefore, it can find the embedded pattern more rapidly.

### Noise and memory robustness

**Fig 4:**
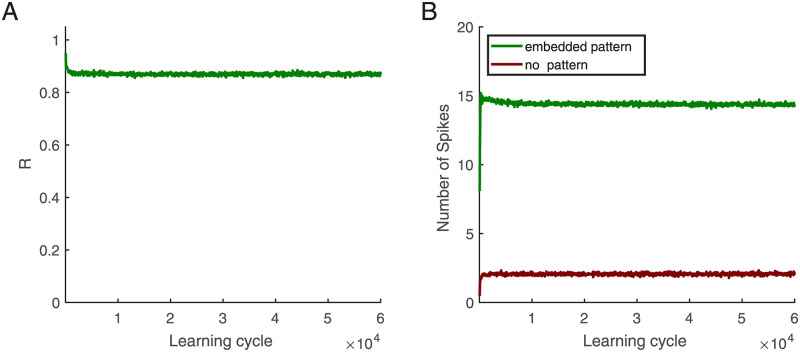
Long term robustness of memory traces. **(A)** *R* as memory criterion versus learning cycle: Without learned patterns in afferents, learning continues, and every 50 cycles, learning is paused, and the learned pattern is placed in the background, then R is computed as a memory criterion. **(B)** Number of spikes versus learning cycle. Neuron elicits more spikes when the embedded pattern is in the afferents (green) than the situation without the pattern (red). Patterns duration is 50 ms, shown in afferents starting from 500 ms, *L* = 15 ms, and this figure is based on an average of 500 simulations.

We wondered if and how the memory for the originally learned pattern decays when synaptic plasticity is present during long periods of random inputs where an already learned pattern does not re-appear. Note that plasticity was switched off when we tested whether it remembers the originally learned pattern. Fig 4A shows that even after 60000 learning cycles, > 80 percent of spikes would still occur during the embedded pattern time (chance level is 0.05). In particular, after dropping to this value *R* remains constant for a period that would correspond to more than 16 hours, with practically no further decay and diffusing in weight vectors. This striking memory persistence can be understood by considering that due to the synaptic scaling inherent the neurons will change synapses until the pre-determined long-term firing rate is achieved also for random patterns (red in Fig 4B). Since the inputs have no structure, the weights mostly become only scaled, which per se cannot erase the selectivity for the learned pattern. When the learned pattern is then again presented to the afferents, the neuron fires additional spikes during the pattern, which leads to a much higher firing rate for the pattern (green in Fig 4B).

This firing with higher rate cannot be considered a real rate code since also with the scaled weights the system remains deterministic. That is, the spikes during the pattern will then still be at precise temporal positions, albeit we will have more spikes than when learned with regularly occurring patterns. Generally, the first spikes in these bursts occur earlier. Furthermore, in many simulations we find the spikes to occur at a similar position as after learning with regularly occurring patterns (not shown). If now the activities of many such neurons would feed again into a subsequent detector neuron it can (as we show for rate based patterns below) still learn to respond to the corresponding pattern, particularly when the pattern is short.

**Fig 5:**
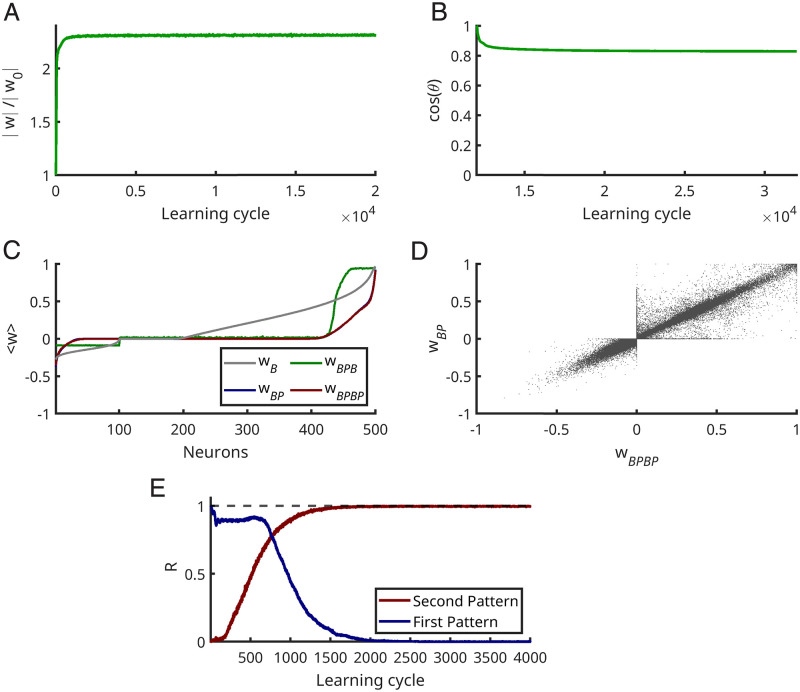
Stability of memory. **(A, B)** Without learned patterns in afferents, learning continues. **(A)** Average of the ratio of the norm of the current weight vector divided by the norm of the learned pattern weight vector (w_0_). **(B)** Average of the cosine between the current weight vector and w_0_. **(C)** Average of weight values in each stage of learning: First neurons learned to fire 2 Hz while there is no embedded pattern in afferents till the learning cycle 2000. *W*_*B*_ (gray line in **(C)**) is the average of sorted weights for this case. Then in each simulation an embedded pattern is present in the afferents for 10000 learning cycles. *W*_*BP*_ (blue line) is the mean of sorted weights obtained after neurons learned the embedded patterns. Next there is again no embedded pattern in afferents for 20000 learning cycles, and *W*_*BPB*_ shows the resulting weight vector at this learning cycle, however, displayed using the ranks from *W*_*BP*_. Finally, there is again the original embedded pattern in the afferents for further 20000 learning cycles; *W*_*BPBP*_ shows the resulting averaged weights with the same sorting. In the mean the sorting is conserved, and the values return to the same size. **(D)** The scatter plot from all simulations demonstrates that most of the weights remain roughly the same, i.e. learning a pattern leads to a fixed point in weight space. **(E)** The neurons first learn the first embedded pattern for the 10000 learning cycles, and then there is another different embedded pattern (second pattern) in afferents for another 10000 learning cycles. This figure shows the R-value for the second 10000 learning cycles. This figure is based on an average of 500 simulations.

The changes between a new weight vector and the initial weight vector can be in amplitude and angle. In order to keep the memory, the learned weight vector should not change its direction in weight space. If the cosine of the angle remains close to one, the neuron would still remember the pattern, while the change in the number of spikes is caused by increasing the norm of a weight vector. As Fig 5A shows the norm of the weight vector indeed changes dramatically while the angle changes are rather insignificant (Fig 5B).

Taken together, we find that when learning is continued with random input the neuron achieves the desired firing rate mostly by scaling its synapses up and then randomly fires in response to the noisy input.

Taking these findings into account both learning and persistence of pattern selectivity can heuristically be understood in combination. Let’s first consider the weight changes caused by stochastic background only. Here, the instability of Hebbian excitatory plasticity drives a subset of weights to large values until the desired number of spikes occurs in the mean (Figs 3A and 5C). Simultaneous Hebbian plasticity of inhibition ensures that global balance is achieved. Then, the neuron is in the fluctuation driven regime, with rather strong excitatory and inhibitory weights which leads to large fluctuations of the membrane potential (Fig 1, blue lines). After achieving this balance further weight changes induced by the stochastic background induce mainly some random walk confined around this fixed point (green line in Fig 5C).

This raises the question if the fixed point of the weights learned from random input alone is too stable to allow for subsequently learning a particular pattern. Fig 3 demonstrates that learning is indeed possible also with this initialization. Intuitively, when only random input is presented the weights perform a random walk around the fixed point, but when a repeating pattern is introduced an additional drift systematically shifts the weights away from this fixed point until the desired number of spikes occur only for the pattern and none in response to the background [30]. Note, that this entails that some of the weights originally acquired from the background (Fig 5C) decay. This weight dynamics continues until the spikes occur only in the period of the repeating pattern. Then the remaining weight changes cause diffusion around this new fixed point (red and blue line in Fig 5C). Hence, also with such an initialization the neuron can learn to fire in response to the embedded pattern. The learning speed, however, is reduced. Fig 3C shows that the R-value approaches one, and Fig 3B that the cosine between the new weight vector and the initial weigh vector changes.

In order to understand why the memory for the learned pattern persists when learning continues without the embedded pattern being present note that the weights for pattern detection are specific for the repeating pattern and at the same time guarantee that the background alone will not elicit spikes. When learning continues without the repeating pattern all weights are mainly scaled up by both the mechanisms of excitatory plasticity, synaptic scaling and the instability of the Hebbian term. In consequence the norm of the weight vector grows (Fig 5A) while its direction is preserved (Fig 5B). Thereby the structure of the learned weights persists.

The trained neuron (with weight vector *W*_*BP*_) has been responding to noisy input for a long time; therefore, weight vectors will be changed to *W*_*BPB*_ (Fig 5C). To show that the embedded pattern is stored in particular synapses, we subsequently present again the embedded pattern and continue learning to determine whether weight vectors (*W*_*BPBP*_) are oriented toward *W*_*BP*_. Fig 5D shows that the most components of weight vectors persist. This illustrates how the memory is protected from being overwritten by noise. It will, however, become overwritten if the trained neuron receives another embedded pattern (Fig 5E).

**Fig 6:**
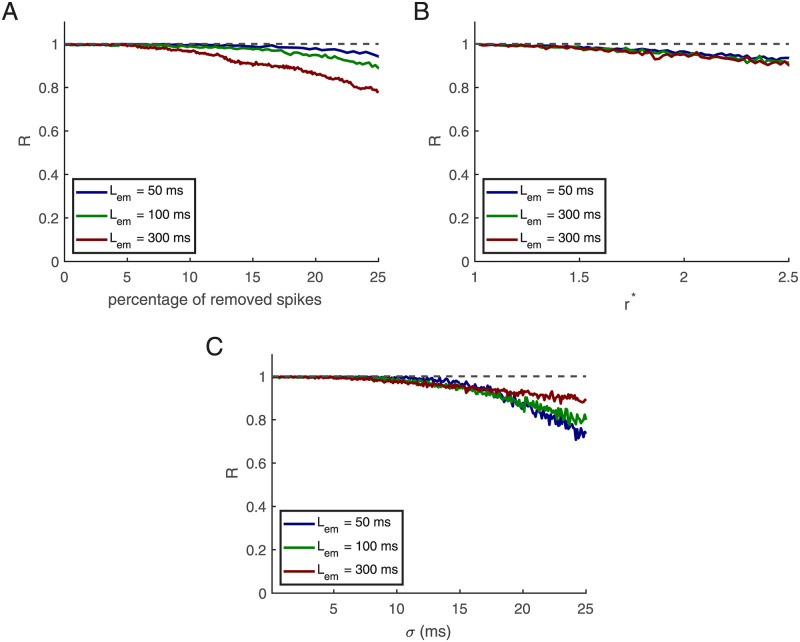
Testing noise robustness. **(A)** Removing spikes. **(B)** Increasing firing rates. **(C)** Jitter noise. Patterns durations are 50, 100, and 300 ms, shown in afferents starting from 500 milliseconds. This figure is based on an average of 500 simulations, in which there are 500 afferents, the length of the training epoch is 1000 ms, and *r*_0_ = 2 Hz.

The plausibility of spike pattern coding depends on its robustness against noise and pattern distortions. For neurons that have learned a particular pattern without noise we examined the dependency of detection performance on three types of perturbations:

**First**: we removed spikes inside the embedded patterns and examined if neurons can still recognize the embedded pattern. Fig 6A shows the robustness against removing spikes inside the embedded pattern.

**Second**: during learning, the neuron receives inhibitory input at a frequency of 20 Hz and excitatory input at a frequency of 5 Hz (ratio 4 to 1). For testing we added *S* random spikes per second to excitatory input neurons and *a* × *S* spikes per second to inhibitory neurons. The new rates becomes $r_{E}^{new}=r_{E}+S$ and $r_{I}^{new}=r_{I}+4S$. Fig 6B shows that the performance R decays when $r_{E}^{new}>10$ Hz. Here we define $r_{}^{*}=\frac{r_{new}}{r}$.

**Third**: in this part, we examine the robustness of detection against jitter noise. For this purpose, we shuffle the times of the spikes in the afferents using a Gaussian distribution with zero mean and *σ* standard deviation. Fig 6C shows that the algorithm is robust until *σ* = 5 ms, and after that, performance starts to decay.

**Fig 7:**
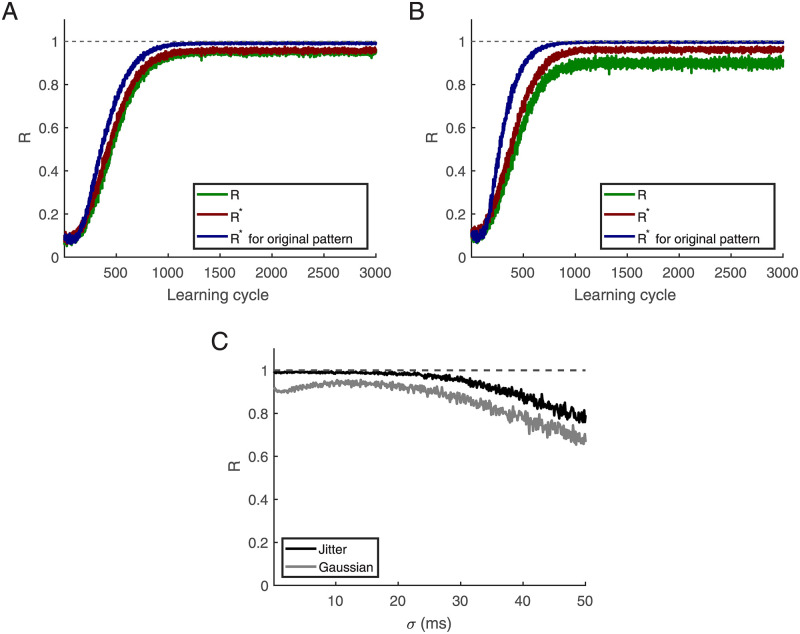
Learning and testing with jittered patterns and Poisson spike rates. **(A)** The training patterns are perturbed by jittering the spikes with a Gaussian distribution with a standard deviation of *σ* = 20 ms and a zero mean. Blue represents testing with the original pattern (*R*), the green line represents testing with jittered data including simulations with no spikes (i.e. *R*), and the red line represents the performanc when testing with jittered data but dropping episodes with no spikes (i.e. R*). **(B)** Learning from Poisson spike rates: the blue line represents *R* when testing with the original pattern, the green line represents testing with Poisson spike rates, including those with no MP spikes (*R*), and the red line shows R* where episodes with no spikes are ignored. **(C)** The black and gray lines represent tests of jittered and Poisson spike rates where the weights were trained with *σ* = 20 ms, respectively. This figure is based on an average of 500 simulations with 500 afferents. The training epoch is 1000 ms, *L*_*em*_ = 50 ms, *L* = 15 ms, and *r*_0_ = 2 Hz.

Next, we wondered if precise patterns are required for self-organizing pattern selectivity. First, we perturbed the training patterns by jittering the spikes according to a Gaussian distribution with mean zero and a standard deviation of 20 ms. We found that this does not hamper learning. Fig 7A shows the R-value in each learning cycle; the blue line is for testing with the original pattern, the green line with the jittered patterns. The red line represents R*, i.e. we dropped contributions to R from the epochs where no spikes at all occur. Then, we converted each afferent’s time code input to rate code input (i.e. Poisson spike rates). For this purpose we first convolved each spike with a Gaussian distribution with a zero mean and a standard deviation of 20 ms. The resulting function is then used as modulated firing rate of a Poisson point process. This results in an ensemble of spatio-temporal patterns based on the original pattern that has the statistics of Poisson processes, including failures and a Fano factor of 1. These distorted patterns are then used for learning and testing. This transformation is leading to a similar result (Fig 7B, the blue line is for testing with the original pattern, the green line for testing with Poisson spike rates including no spikes, the red when epochs with no spikes are dropped).

It turns out that with the corresponding weights pattern detection becomes more robust with respect to jitter noise (Fig 7C, the black and gray lines are for testing with different *σ* for jittered and Poisson spike rates, respectively). These results demonstrate that codes based on temporal rate modulations and spatio-temporal pattern detection by individual neurons are compatible.

### Diversification by pre-synaptic hetero-synaptic plasticity

**Fig 8:**
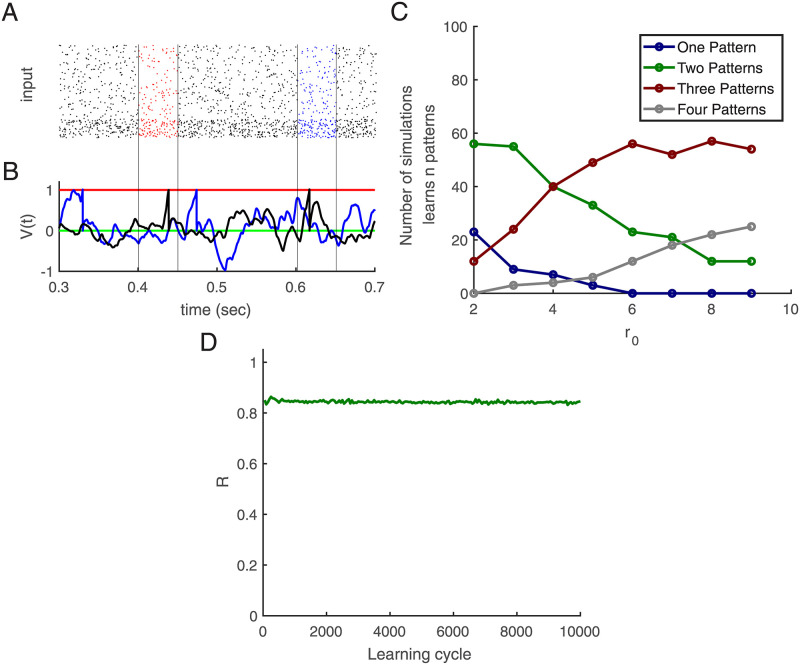
Learning more than one embedded pattern. **(A)** Input activity in the raster plot, five hundred afferents (80% excitatory and 20% inhibitory) send inputs to one post-synaptic neuron. The excitatory neurons’ rate is 5 Hz, and the inhibitory neurons’ rates are 20 Hz. There are two random embedded patterns of length 50 ms in the red and blue areas (between black vertical lines), *r*_0_ = 4 Hz, and the length of the training epoch is 1000 ms. **(B)** Membrane potential versus time. The black trace shows that after learning the neuron responds to both embedded patterns. The green line is for resting potential, red is for threshold, blue is for before learning, and black is for after learning. **(C)** During learning, all embedded patterns are in the epoch, and we compute the percentages of neurons that can detect only one of the patterns (blue), two (green), three (red), and four of them depending on the target rate *r*_0_. This figure is based on an average of 100 simulations with 500 afferents. The training epoch is 1000 ms, *L*_*em*_ = 50 ms, *L* = 15 ms. **(D)** First, the neuron learns two embedded patterns, and then *R* as a memory criterion vs. learning cycle is computed: Learning continues without learned patterns in afferents, and every 50 cycles, learning is paused, and the learned patterns are placed in the background, then R is computed as a memory criterion. There are two 50 ms embedded patterns, shown in afferents starting from 400 ms and 800 ms, *L* = 15 ms, and this figure is based on an average of 500 simulations. *r*_0_ = 4 Hz.

In the approach presented so far a single neuron can become a detector for more than only one pattern, along the lines of the Tempotron [9] (Fig 8A, 8B and 8C). Its activity would then, however, obscure which individual pattern was present at which time. The number of patterns a single neuron can learn depends on the desired firing rate. As shown in Fig 8C, 60 percent of the ensemble learns two patterns when *r*_0_ is two, but when it is nine, almost 50 percent of the ensemble learns at least three patterns (Note there are for independent embedded patterns.). Here the memory for the case of two embedded patterns and a single post-synaptic neuron is tested too. When synaptic plasticity is active throughout extended periods of random inputs when previously learned patterns do not reappear, memory for them decays. We turned off plasticity when we investigated whether it recalls the first learned patterns. Fig 8D indicates that even after 10000 learning cycles, more than 80% of spikes would still occur within the embedded pattern duration (chance level is 0.1). Here, we consider R as the summation of the R-values for patterns one, *R*_1_, and two, *R*_2_. Therefore *R* = *R*_1_ + *R*2.

For a faithful representation of a sequence of patterns, as e.g. the phonemes in spoken human language, it is therefore desirable that different neurons in a network become specialized for different subsets of patterns such that as a whole a network represents precisely the which and when of patterns in a sequence. If successful, such a system could, e.g., be used for unsupervised speech recognition [16].

As a first step into this direction we wondered which realistic synaptic mechanism might enforce the specialization of different neurons in a network for different pattern sets. As a simple example we consider several target neurons that receive input from the same set of input neurons. We found that already synaptic competition induced by pre-synaptic hetero-synaptic plasticity [26] yields sufficient selectivity for different patterns in such an ensemble of target neurons such that identity and order of patterns are represented faithfully (for details of the computational implementation see Materials and methods).

To quantify the performance, we use the rank of a matrix where each row represents one of the neurons, and each column one of the patterns. The matrix element in each row is set to 1 if the corresponding neuron is active for that pattern, and to 0 otherwise. The rank of this matrix provides the number of linearly independent row vectors. When it meets the number of the different patterns in a given stimulus the population of neurons faithfully represents the presence and the order of the patterns. Therefore, we introduce the ratio of the rank of this matrix to the number of patterns as performance measure Ω.

**Fig 9:**
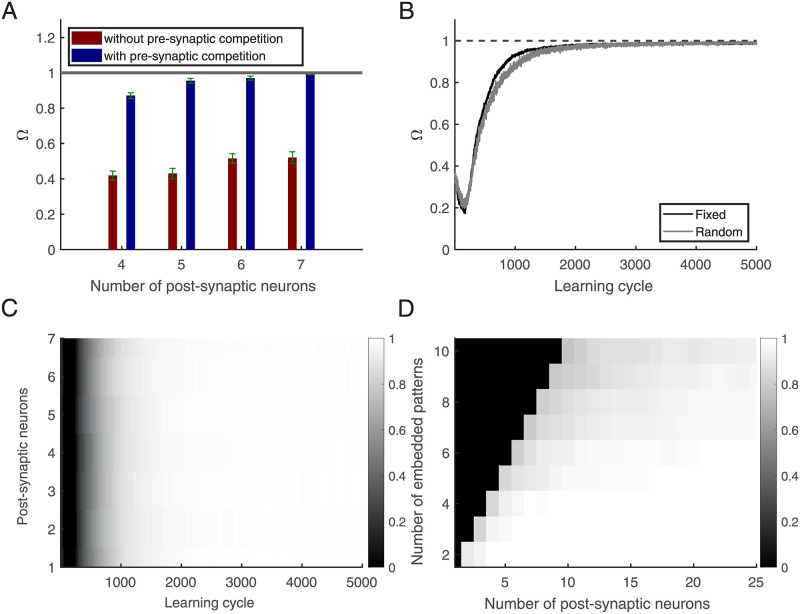
Self-supervised neural networks. **(A)** The system’s Ω (rank/4) versus the number of post-synaptic neurons after learning. **(B)** Ω versus learning cycle (There are 7 post-synaptic neurons). Black line: Each pattern is shown in each learning cycle in a fixed position. Gray line: Each pattern is shown in the epoch with the probability of 0.2 at a random place. **(C)** Each row shows the mean R values for the case of 7 post-synaptic neurons. **(D)** Ω: number of post-synaptic neurons to separate different numbers of embedded patterns (Ω is computed when the number of post-synaptic neurons equals or exceeds the number of embedded patterns.) **(A)**, and **(D)** are based on an average of 50 simulations. **(B)** and **(C)** are based on an average of 500 simulations. In all simulations, the epoch length is 1000 ms, the embedded patterns’ duration is 50 ms, *L* = 15 ms, and *r*_0_ = 2 Hz. In **(A)**, **(B)**, and **(C)**, there are 4 different independent embedded patterns in each simulation.

As an example, we trained networks of different sizes with four patterns where always all patterns were present in each training epoch. We found that the pre-synaptic competition leads to selectivity for all four patterns as soon as 7 post-synaptic neurons were present. In contrast, without this pre-synaptic competition, separation does not become complete (Fig 9A).

We then tested the persistence of learned selectivities (with 7 neurons) when learning is continued with random input. We tested performance (with plasticity switched off) and found practically no decay of Ω even when learning was continued 10X longer than it takes for learning all 4 patterns.

This result motivated us to test if learning a set of patterns is possible from learning epochs that contain only subsets of all patterns. First results indicate that such incremental learning is indeed possible. As an example, we considered 4 embedded patterns and 7 post-synaptic neurons. Each embedded pattern has a 0.2 probability of being present in each learning cycle in the epoch. Note that thereby some learning cycles have no embedded pattern in the epoch. Also, we randomly chose locations from ([85, 85 + *L*_*em*_), [175, 175 + *L*_*em*_), …[895, 895 + *L*_*em*_] ms) to put the embedded patterns in them. As Fig 9B shows the relative rank Ω goes to one, indicating that patterns can be learned also incrementally and the R value goes to one for all post-synaptic neurons Fig 9C.

**Fig 10:**
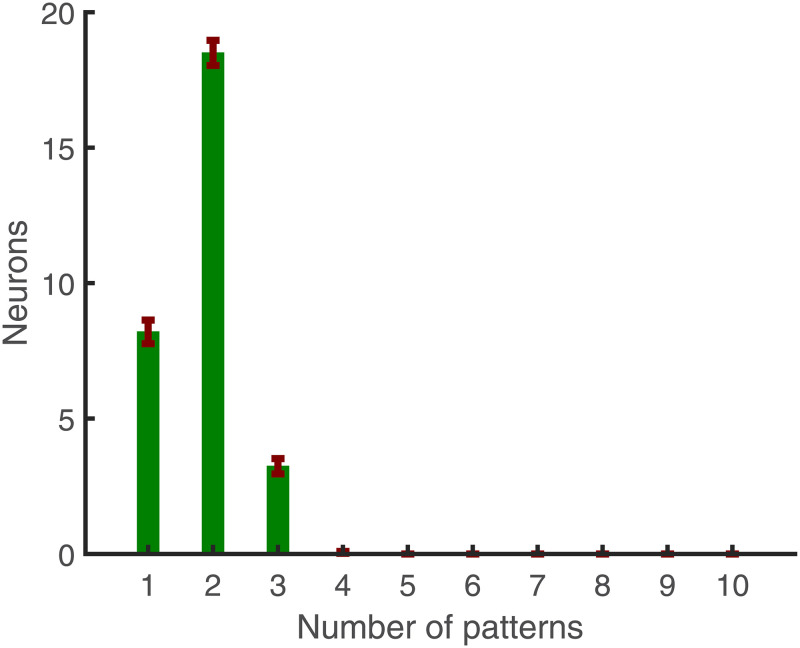
Contingent of neurons responding to multiple embedded patterns. Average count of post-synaptic neurons versus the number of embedded patterns detected. This figure is based on an average of 50 simulations with epoch length 1000 ms, 30 post-synaptic neurons, and 10 independent embedded patterns, having a duration of 50 ms each, (*L* = 15 ms, and *r*_0_ = 2 Hz.)

We then tested how many post-synaptic neurons are required to separate different numbers of embedded patterns. Fig 9D shows a linear increase in the number of post-synaptic neurons necessary for selecting different numbers of embedded patterns (where Ω ⩾ 0.96). However, most of the post-synaptic neurons respond to only two of the embedded patterns. Here we look at which post-synaptic neuron fires for which patterns and count them. As shown in Fig 10, most neurons respond to a few embedded patterns; on average, 8 respond to only one, 19 to two, and 3 to three embedded patterns.

## Discussion

Neurons respond faithfully to input sequences [31]. That is, they are rather deterministic devices and with suitable synaptic efficacies, individual neurons can, in principle, serve as detectors for specific spatio-temporal input spike patterns. This opens the possibility that coding and computation in brains are at least in part based on temporally precise action potentials.

In the past, this hypothesis has been investigated in simple integrate and fire models. Supervised learning rules were proposed that enable neurons to signal the presence of a pattern [9] and to fire at predefined time points during a specific pattern [10, 13, 32]. It was further demonstrated that relatively weak supervision can be sufficient for learning the synaptic weights for pattern detection [16]. Here, only knowledge about the number of pattern occurrences is needed for the specialization of a neuron. While a learning rule for this ‘aggregate label learning’ was rigorously derived, the proposed biological realization suffers from several rather unrealistic assumptions. In particular, excitatory and inhibitory plasticity are not treated separately, and Dale’s law was not observed. When the signs of synapses may change, this has also a negative impact on the excitatory and inhibitory balance. For some patterns, there may be only few inhibitory neurons remaining in the system after learning. When there is a lack of inhibition, however, the potential can take a value close to the threshold, increasing the chance of getting random spikes outside the embedded patterns. Also, a selection criterion was used, by which independently of their sign, only the largest 10% of changes were taken into account, for which no realistic interpretation was provided.

Unsupervised learning of spatio-temporal input spike patterns has been investigated mostly in the context of spike-timing dependent plasticity (STDP) [33–35]. The model in Masquelier 2018 [36] discusses the ability of STDP of excitatory synapses to make neurons become spike pattern detectors. It requires quite careful tuning of parameters. A model closer in spirit to the present one can be found in a rather brief technical report [37]. It combines some (calcium based additive) homeostatic plasticity with a caricature of correlation based learning solely for excitatory synapses. It is demonstrated to mimic STDP and admittedly also requires careful choice of parameters. Most importantly, because of the lack of inhibition both previous models cannot exhibit balance of excitation and inhibition and therefore can neither be expected to be robust against perturbations nor to be stable with respect to continuous learning without patterns.

Therefore, it remains an open question if the synaptic plasticity mechanisms present in real neuronal networks support a robust coding scheme that is based on spatio-temporal patterns of spikes.

We found that a combination of membrane potential dependent Hebbian mechanisms, hetero-synaptic competition, and synaptic scaling indeed makes individual neurons sensitive for statistically dominant spatio-temporal patterns in their afferents without any supervision. The hetero-synaptic competition implies a threshold for long-term depression (LTD) depending on the ensemble of plasticity signals, which could be related to the Bienenstock-Cooper-Munroe (BCM) rule [38]. Along the same line the synaptic scaling regulates weight changes such that with too high post-synaptic firing rates the synapses decay. In contrast, the set of mechanisms considered here can not reproduce STDP, since mono-synaptic LTD-mechanisms are not taken into account.

Performance is shown to be robust to temporal jitter, missing spikes, and additional noise. In particular, also spatio-temporal patterns consisting of Poisson spike rate modulations are captured surprisingly well by the proposed plasticity mechanisms and lead to robust detection (Fig 7B and 7C).

The proposed combination of learning mechanisms yields a detailed balance of excitation and inhibition where this is possible: during the learned pattern. This fits nicely to experimental observations [22] revealing a negative correlation between excitatory and inhibitory inputs. Outside the pattern, global balance is achieved [24].

Balance is a natural consequence of Hebbian mechanisms when they are simultaneously present in both excitatory and inhibitory synapses, and the otherwise unstable growth of excitatory efficacies is constrained. While this has been noted before [39], we here show that Hebbian plasticity can select synaptic efficacies that make neurons detectors for spatio-temporal patterns when realistic constraints are taken into account. In particular, we found that a subtle interplay of the instability of Hebbian mechanisms for excitatory synapses and synaptic scaling enforces a local imbalance during the learned patterns which leads to specific and temporally precise spike responses.

The excitatory-inhibitory balance protects memory for rate patterns in neural networks [39, 40]. Our simulations now demonstrate that also the memory for spike patterns is protected from being overwritten by noise (Figs 4, 5D and 5E).

After successful learning of patterns, the random background input does not lead to spikes in individual neurons. When then plasticity is continuously present for a long time during which only random inputs are present, the input weights mainly become scaled up until the desired number of spikes is reached, i.e., the plasticity mechanisms considered here are consistent with synaptic scaling [20]. Obviously, scaling alone preserves the memory for the learned patterns, which will then induce far more spikes than the background. In other words, the plasticity mechanisms discussed here lead to a very long memory persistence already in a single neuron such that selectivity is preserved and sensitivity becomes even enhanced. We tested the memory persistence also in groups of neurons that specialized for subsets of the input patterns via pre-synaptic hetero-synaptic plasticity. Also here, we observed practically no decay.

This finding suggests that in groups of neurons incremental learning of sets of patterns should be possible, wherein each successive training epoch only a subset or even none of all patterns is present. We could confirm this hypothesis for simple cases; however, the question if the combination of plasticity mechanisms discussed here indeed provides a solution for the notorious stability-plasticity dilemma [25] in spatio-temporal pattern learning will require more systematic investigations which go beyond the scope of this paper.

The fact that biologically realistic plasticity mechanisms can support the self-organization of spatio-temporal pattern detection by individual neurons underlines the possibility that such temporal codes are indeed present in nervous systems. Particularly the ability to learn patterns underlying Poisson spike rates demonstrates that such a temporal coding scheme can be consistent with rate codes. We speculate that this transformation of temporally modulated rates to spike pattern codes could explain the increase in sparsity observed in the early visual cortex when natural contexts are included [41] as well as the extreme sparsity of activations in higher cortical areas.

## Materials and methods

### Neuron model

In this work, we employ the leaky integrate and fire (LIF) model. The dynamic of the membrane potential of a single neuron *V*(*t*) which is receiving current from *N* afferents is:

$$\begin{array}{c} \tau_{m}\frac{d}{dt}V\left(t\right)=-V\left(t\right)+R_{m}I_{ext}\left(t\right) \end{array}$$ (3)

where *τ*_*m*_ and *R*_*m*_ are time constant and resistance of the membrane, respectively. Whenever the neuron arrives at or passes the threshold it evokes spike output and resets to resting potential. Resting potential is zero in this study, and *I*_*ext*_ is the external current from excitatory (E) and inhibitory (I) afferents:

$$\begin{array}{c} I_{j}\left(t\right)=\sum_{i=1}^{N_{E}}w_{ji}^{E}\sum_{t_{i}^{l}<t}^{}K^{E}\left(t-t_{i}^{l}\right)-\sum_{i=1}^{N_{I}}w_{ji}^{I}\sum_{t_{i}^{l}<t}^{}K^{I}\left(t-t_{i}^{l}\right) \end{array}$$ (4)

where $w_{ji}^{E,I}$ represent the respective *i*^′^*th* afferent’s excitatory and inhibitory synaptic strength to output neuron *j*. *N*_*E*_ and *N*_*I*_ are the numbers of excitatory and inhibitory afferents, respectively (*N* = *N*_*E*_ + *N*_*I*_). Note that at time $t_{i}^{l}$, there is a spike in afferent *i*, and at this time, afferent *i* starts to send input to the post-synaptic neuron *j* amounting to *w*_*ji*_ × *K*. The shape of the kernel is an alpha-function with the following equation:

$$\begin{array}{c} K\left(t-t_{i}^{l}\right)=I_{norm}[\text{exp}(-\frac{t-t_{i}^{l}}{\tau_{r}})-\text{exp}(-\frac{t-t_{i}^{l}}{\tau_{d}})]\theta \left(t-t_{i}^{l}\right) \end{array}$$ (5)

where *θ* is the Heaviside step function. *τ*_*r*_ and *τ*_*d*_ are time constants of synaptic current, which are different for excitatory and inhibitory synapses. $I_{norm}=\frac{\eta^{\eta /\eta -1}}{\eta -1}$ normalises *K* to unit amplitute, where $\eta =\frac{\tau_{d}}{\tau_{r}}$.

### Learning algorithm

**Fig 11:**
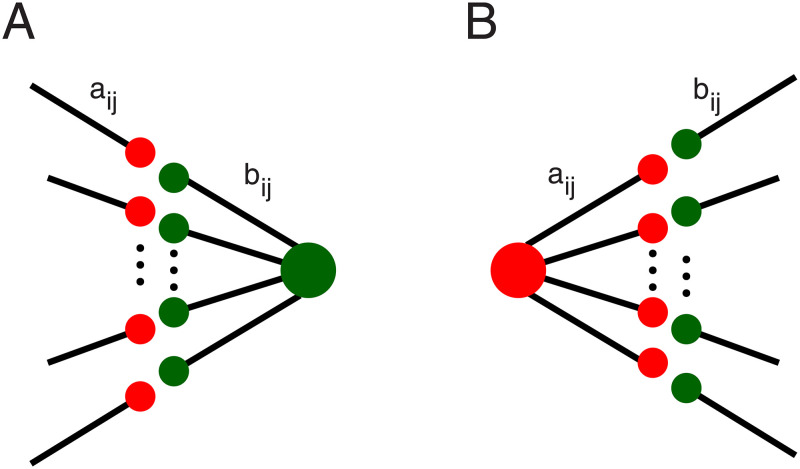
Illustration of synaptic competition induced by hetero-synaptic plasticity. **(A)** Competition induced by post-synaptic hetero-synaptic plasticity. Excitatory pre-synaptic neurons i target a post-synaptic neuron j. We assume that the resources required for increasing the post-synaptic components *b*_*ij*_ of the weights *w*_*ji*_ = *a*_*ij*_*b*_*ij*_ are limited and therefore distributed in a competitive manner. That is, we assume hetero-synaptic plasticity where afferent synapses that receive large eligibility signals *ε*_*ij*_ increase their efficacy while synapses that receive weaker (but for excitatory synapses always positive) signals will instead weaken. **(B)** Pre-synaptically induced competition is assumed to follow the same principle. The signals for the changes of the pre-synaptic components *a*_*ij*_, however, are assumed to depend on the realized amount of potentiation at the post-synaptic side, i.e., to take the post-synaptic hetero-synaptic competition into account. We found that this choice is more parameter tolerant and yields more robust memory than a symmetric version where pre- and post-synaptic hetero-synaptic plasticity are based on the same eligibility signal which, however, can also realize self-organized spike pattern detection (not shown).

We assume that a synapse’s efficacy is the product of pre- and post-synaptic components.

$$\begin{array}{c} w_{ji}=a_{ij}b_{ij} \end{array}$$ (6)

where *a*_*ij*_ is provided from the pre-synaptic neuron *i*, and *b*_*ij*_ is provided by the post-synaptic neuron *j* (Fig 11). If we have *N* pre-synaptic neurons (*i* = 1, …, *N*) and *M* post-synaptic neurons (*j* = 1, …, *M*)

$$\begin{array}{c} \frac{dw_{ji}}{dt}=a_{ij}\frac{db_{ij}}{dt}+\frac{da_{ij}}{dt}b_{ij}. \end{array}$$ (7)

Here, Dale’s law is imposed: when *δa* and *δb* would change the sign of *a* and *b*, respectively, we set them to zero. We assume that correlations between pre-synaptic input and post-synaptic membrane potential drive weight changes. By considering only the spike at time $t_{i}^{l}$ the correlation between *i*^′^*th* afferent and the *j*^′^*th* post-synaptic neuron’s potential *V*_*j*_(*t*) is:

$$\begin{array}{c} \vartheta_{ij}^{*l}\left(t\right)=K\left(t-t_{i}^{l}\right)\left\{{\left[V_{j}\left(t\right)-V_{0}\right]}_{+}+q{\left[V_{j}\left(t\right)-V_{0}\right]}_{-}\right\} \end{array}$$ (8)

where *V*_0_ is the modification threshold [42] that is set to zero in all simulations of this study. *q* is zero for excitatory afferents and 1 for inhibitory ones. In every time step, all *n*_*i*_ spikes in the *i*^′^*th* afferent are used for updating the weights.

$$\begin{array}{c} \vartheta_{ij}\left(t\right)=\sum_{l=1}^{n_{i}}\vartheta_{ij}^{*l}\left(t\right). \end{array}$$ (9)

As a result, we compute the correlations for all spikes *l* and integrate them to determine the eligibility *ε* [16] via

$$\begin{array}{c} \tau_{\varepsilon }\frac{d\varepsilon_{ij}\left(t\right)}{dt}=-\varepsilon_{ij}\left(t\right)+\vartheta_{ij} \end{array}$$ (10)

which is the basic signal for weight changes at synapse (*i*, *j*).

For inhibitory synapses weight changes are set to be simply proportional to *ε*. For excitatory synapses changes are assumed to depend only on the positive part [*ε*_*ij*_]_+_ which mimics the characteristics of the NMDA receptor [17–19]. Also for excitatory weights we take synaptic scaling into account. That is, neurons tend to fire and to implement a specific firing rate *r*_0_ that is genetically determined [20, 29]. That is, if a post-synaptic neuron’s long-term firing rate $r_{j}^{*}$ is less than the desired *r*_0_, it scales its afferent excitatory weights up, and if it is more than the desired *r*_0_, it scales its afferent excitatory weights down. The long-term firing rate $r_{j}^{*}$ is determined by

$$\begin{array}{c} \tau_{r^{*}}\frac{dr_{j}^{*}}{dt}=-r_{j}^{*}+\sum_{t_{i}^{l}}^{}\delta \left(t-t_{i}^{l}\right) \end{array}$$ (11)

where $\tau_{r^{*}}$ is the time constant for the long-term firing rate. Changes of synapses are subject to limitations of the material provided by the respective pre and post-synaptic neurons (e.g., release sites, vesicles, receptor densities). Plausibly, this leads to competition for changes of different synapses, which affects the respective pre- and post-synaptic components a and b differently. In the current approach we include pre- and post-synaptic competition only for the changes of excitatory weights thereby modeling pre- and post-synaptic versions of hetero-synaptic plasticity [21, 26, 43, 44].

#### Single post-synaptic neuron

To compute weight change (Eq (7)) when there is only a single post-synaptic neuron (*j* = 1), we have *b*_*i*1_ ≕ *b*_*i*_, *ε*_*i*1_ ≕ *ε*_*i*_, and *a*_*i*1_ ≕ *a*_*i*_. For simplicity we here set *a*_*i*_ = 1 and *δa*_*ij*_ = 0 (for the full version see next section).

Here inhibitory synapses are changed by

$$\begin{array}{c} \tau_{I}\frac{db_{i}^{I}}{dt}=-b_{i}^{I}+c^{I}\varepsilon_{i}^{} \end{array}$$ (12)

where *τ*_*I*_ is the time constant for inhibitory synapses and *c*^*I*^ the learning rate.

For excitatory synapses we subtract the mean of the plasticity signals [*ε*_*i*_]_+_, to mimick post-synaptic hetero-synaptic plasticity:

$$\begin{array}{c} \tilde{\varepsilon_{i}}=\varepsilon_{i}-\frac{1}{N}\sum_{i=1}^{N}\varepsilon_{i}. \end{array}$$ (13)

Note that this does not imply strict normalization since Dale’s law prevents negative changes that otherwise would turn excitatory synapses to inhibitory synapses. The excitatory afferents’ weights then change according to the following equation:

$$\begin{array}{c} \tau_{E}\frac{db_{i}^{E}}{dt}=-b_{i}^{E}+c^{E}{\tilde{\varepsilon }}_{i}^{}+\alpha b_{i}^{E}\left(r_{0}-r\right) \end{array}$$ (14)

where *τ*_*E*_ is the time constant for excitatory synapses and *c*^*E*^ the learning rate. We consider *c*^*E*^ less than *c*^*I*^; therefore, inhibitory neurons adapt faster to ensure balance wherever possible (globally and detailed), and excitation can not conflict with the relatively slow limiting mechanism of synaptic scaling.

#### More than one post-synaptic neuron

When there is more than one post-synaptic neuron each afferent has different eligibility for each post-synaptic neuron (Eq (9)). Therefore *a*_*ij*_ needs to be taken into account (Eq (7)).

For inhibitory synapses we use:

$$\begin{array}{c} \begin{array}{c} \tau_{I}\frac{db_{ij}^{I}}{dt}=-b_{ij}^{I}+c^{I}\varepsilon_{ij}^{}\\ \tau_{I}\frac{da_{ij}^{I}}{dt}=-a_{ij}^{I}+c^{I}\varepsilon_{ij}^{} \end{array} \end{array}$$ (15)

As for single target neurons *j* the changes of excitatory synapses are subject to post-synaptic hetero-synaptic plasticity that we realize by subtracting the mean of eligibilities:

$$\begin{array}{c} {\tilde{\varepsilon }}_{ij}=\varepsilon_{ij}-\frac{1}{N}\sum_{i=1}^{N}\varepsilon_{ij}. \end{array}$$ (16)

With this the post-synaptic components change by

$$\begin{array}{c} \begin{array}{c} \tau_{E}\frac{db_{ij}^{E}}{dt}=-b_{ij}^{E}+c^{E}{\tilde{\varepsilon }}_{ij}+\alpha b_{ij}^{E}\left(r_{0}-r\right). \end{array} \end{array}$$ (17)

We assume that also pre-synaptic neurons have finite resources for increasing their contributions to synaptic efficacies (*a*_*ij*_), as e.g., release sites or vesicle densities. Fig 11 illustrates this pre- and post-synaptic competition. We restrict the competition to excitatory neurons that up-regulate synapses because this requires resources, whereas decreases of efficacies may even release resources. To apply the competition, we first identify the signals that would scale up excitatory synapses. The pre-synaptic competition then is implemented by subtraction of the mean

$$\begin{array}{c} B_{ij}={\left[{\tilde{\varepsilon }}_{ij}\right]}_{+}-\frac{\theta (\sum_{j}\theta \left({\left[{\tilde{\varepsilon }}_{ij}\right]}_{+}\right)-1)}{\sum_{j}\theta \left({\left[{\tilde{\varepsilon }}_{ij}\right]}_{+}\right)+\zeta }\sum_{j=1}^{M}{\left[{\tilde{\varepsilon }}_{ij}\right]}_{+} \end{array}$$ (18)

where *θ* is a Heaviside function, *ζ* is a small number (*ζ* <<1), and M is the number of post-synaptic neurons. Therefore

$$\begin{array}{c} \begin{array}{c} \tau_{E}\frac{da_{ij}^{E}}{dt}=-a_{ij}^{E}+B_{ij}+\alpha a_{ij}^{E}\left(r_{0}-r\right). \end{array} \end{array}$$ (19)

#### Simulation details

For simplification and efficiency of simulations, we consider changes over epochs *e* with duration *T* ms instead of applying weight changes in each integration time step. Thereby Eq (8) is replaced by:

$$\begin{array}{c} \vartheta_{ij}^{l}=\int_{0}^{T}dtK\left(t-t_{i}^{l}\right)\left\{{\left[V_{j}\left(t\right)-V_{0}\right]}_{+}+q{\left[V_{j}\left(t\right)-V_{0}\right]}_{-}\right\} \end{array}$$ (20)

and the differential equations of the form

$$\begin{array}{c} \tau \frac{dy}{dt}=-y+x \end{array}$$ (21)

change to a moving average

$$\begin{array}{cc} y(e+1)= & \gamma y\left(e\right)+\left(1-\gamma \right)\hat{x}\left(e\right). \end{array}$$ (22)

where $\hat{x}(e)$ is the sum of contributions of *x* in each epoch *e* and *γ* ≃ 1 − *T*/*τ*.

In all simulations, there are 500 afferents (80% excitatory and 20% inhibitory), and the learning epoch length is 1000 ms. Afferent and embedded pattern spikes are generated randomly with Poisson point processes in which the excitatory rate (*r*_*E*_) is 5 Hz, and the inhibitory rate (*r*_*I*_) is 20 Hz. Except for Fig 9B (gray line), all embedded patterns are in each epoch in every learning cycle.

In the network model, the initial synaptic efficacies of *a*_*ij*_ and *b*_*ij*_ are chosen from a Gaussian distribution with a mean of 0.1 and standard deviations of 10^−2^ (negative numbers are set to zero). In the single post-synaptic neuron model, they are chosen from a Gaussian distribution with a mean of 10^−2^ and standard deviations of 10^−3^ (negative numbers are set to zero).

Synaptic scaling depends on firing rates averaged over long times. Since we perform on-line learning we use a low pass filter such that the rate estimation *r*_*j*_(*c* + 1) of post-synaptic neuron *j* used for learning in epoch *e* + 1 is a running average of the actual rates $\hat{r}$ in previous epochs:

$$\begin{array}{cc} r_{j}\left(e+1\right)= & \gamma^{*}r_{j}\left(e\right)+\left(1-\gamma^{*}\right){\hat{r}}_{j}\left(e\right) \end{array}$$ (23)

The parameter γ* is 0.9 for all figures which corresponds to a time constant of 10s. While much smaller values of this parameter do not change the asymptotic results this value was found to yield more rapid convergence.

Note that plasticity is not instantaneous but depends on an accumulation of signals for weight changes over some time which here is termed ‘eligibility’. We implement also this by a low pass filter:

$$\begin{array}{c} \begin{array}{cc} \varepsilon_{ij}\left(e+1\right)= & \gamma \varepsilon_{ij}\left(e\right)+\left(1-\gamma \right){\hat{\vartheta }}_{ij}\left(e\right). \end{array} \end{array}$$ (24)

**Fig 12:**
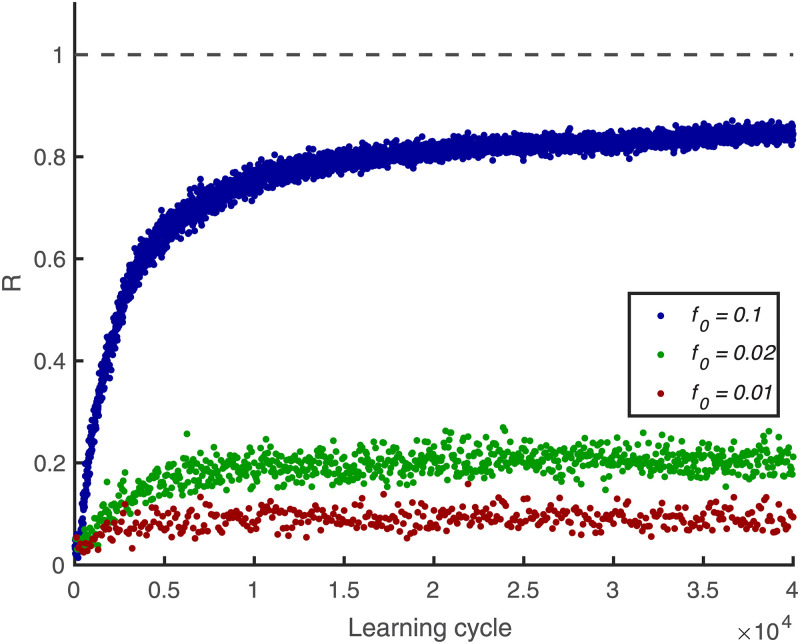
Learning performance for embedded patterns presented at different rates. Learning performance R versus learning cycle, for *L* = 15 ms. A 50 ms pattern is embedded between 500 and 550 ms and shown each 10 cycles (*f*_0_ = 0.1), 50 cycles (*f*_0_ = 0.02), and 100 cycles (*f*_0_ = 0.01). R is an average of 100 simulations, in which there are 500 afferents, and the length of the training epoch is 1000 ms. The desired firing rate is *r*_0_ = 2 Hz.

Initial conditions are *r*_*j*_(0) = 0 and *ε*_*ij*_(0) = 0. The parameter *γ* is 0.99 for all figures corresponding to a time constant of ≃ 100s. This parameter limits learn-ability of patterns that are presented only rarely. E.g. for the value used in this paper learning becomes difficult when a pattern is shown only every hundredths epoch of 1000ms length (Fig 12).

Because synaptic strength cannot become arbitrarily large due to the synapses’ structure and other constraints, we cut weights changes that would carry excitatory synapses out of the bounds which are set to plus one.

Besides, Dale’s rule dictates that excitatory and inhibitory synapses cannot turn into each other; therefore, we assume synaptic weights to become zero if weight changes would turn their kind during the learning. As a result, subtracting the mean value in equations Eqs (13) and (18) will not change a synapse’s type (Eq (7)). To numerically integrate Eq (3), we use Euler method with Δ*t* = 0.1 ms. The parameters are found in Table 1.

**Table 1:** List of parameters.

Symbol	Description	Value
*τ*_*m*_	membrane time constant	15 ms
*R*_*m*_	membrane resistance	1
$\tau_{r}^{e}$	Rise time of excitatory currents	0.5 ms
$\tau_{r}^{i}$	Rise time of inhibitory currents	1 ms
$\tau_{d}^{e}$	Decay time of excitatory currents	3 ms
$\tau_{d}^{i}$	Decay time of inhibitory currents	5 ms
*r*_*E*_	Rate of excitatory neurons	5 Hz
*r*_*I*_	Rate of inhibitory neurons	20 Hz
N	Number of pre-synaptic neurons	500
*N*_*E*_	Number of pre-synaptic excitatory neurons	400
*N*_*I*_	Number of pre-synaptic inhibitory neurons	100
*c* ^*I*^	Inhibitory learning rate	10^−3^
*c* ^*E*^	Excitatory learning rate	0.9 × 10^−3^
Δ*t*	time step	0.1 ms
*α*	scaling factor	0.01
*β*	scaling coefficient	0.9 × 10^−4^

Note that in the epoch approach, we implement the following equations, and the continuous version can be obtained by linearization. Therefore:

$$\begin{array}{c} \begin{array}{c} \delta w_{ij}=a_{ij}\delta b_{ij}+\delta a_{ij}b_{ij}+\delta a_{ij}\delta b_{ij} \end{array} \end{array}$$ (25)

**Single post-synaptic neuron**:

Inhibitory synapses:

$$\begin{array}{c} \begin{array}{c} \delta b_{i}^{I}=c^{I}\varepsilon_{i}^{} \end{array} \end{array}$$ (26)

Excitatory synapses:

$$\begin{array}{c} \delta b_{i}^{E}=\left(1-\beta \right)b_{i}^{E}exp\left(\alpha \left(r_{0}-r\right)\right)+c^{E}{\tilde{\varepsilon }}_{i}-b_{i}^{E} \end{array}$$ (27)

**More than one post-synaptic neuron**

Inhibitory synapses:

$$\begin{array}{c} \begin{array}{cc} \delta b_{ij}^{I}= & c^{I}\varepsilon_{ij}^{}\\ \delta a_{ij}^{I}= & c^{I}\varepsilon_{ij}^{} \end{array} \end{array}$$ (28)

Excitatory synapses:

$$\begin{array}{c} \begin{array}{cc} \delta b_{ij}^{E}= & \left(1-\beta \right)b_{ij}^{E}exp\left(\alpha \left(r_{0}-r\right)\right)+c^{E}{\tilde{\varepsilon }}_{ij}-b_{ij}^{E}\\ \delta a_{ij}^{E}= & \left(1-\beta \right)a_{ij}^{E}exp\left(\alpha \left(r_{0}-r\right)\right)+c^{E}B_{ij}-a_{ij}^{E} \end{array} \end{array}$$ (29)

where *β* is scaling coefficient.
